# Supplementary material for: Are carbon emissions trading and green financial instruments synergistic? -Comprehensive quantitative research based on content analysis
Source: PLoS One. 2024 Mar 7;19(3):e0298601. doi: 10.1371/journal.pone.0298601 (PMC10919601; doi:10.1371/journal.pone.0298601)
Supplement: S1 Data — (ZIP) [file pone.0298601.s001.zip › Data/地级市Z_F工作报告.zip!/╣π╢1⁄2/╖≡╔╜╩╨/└┤╫╘╣1⁄2╓┌║┼í╛┬φ┐╦╩2╛▌═°í┐_2014.docx]

各位代表：

　　现在，我代表佛山市人民政府向大会报告政府工作，请予审议，并请政协各位委员和其他列席人员提出意见。

2013年工作回顾

　　2013年，是全面贯彻党的十八大精神的开局之年。市政府认真落实中央和省委、省政府的决策部署，在市委的正确领导和市人大、市政协的监督支持下，坚持以科学发展观为统领，以提高经济发展质量和效益为中心，着力稳增长、调结构、促改革、惠民生，顺利完成市第十四届人大三次会议确定的各项任务。全市实现地区生产总值7010.17亿元，增长10.0%；来源于佛山市财政收入1486.70亿元，增长17.84%，其中地方公共财政预算收入437.88亿元，增长14.01%；预计城镇居民人均可支配收入38040元，增长10.0%；农村居民人均纯收入17503元，增长11.6%；居民消费价格指数102.5；常住人口自然增长率7.86％。。

　　一年来，我们主要做了以下工作：

　　一、着力推进经济结构调整，经济发展质量效益提升

　　经济发展稳中向好。全年经济运行总体平稳，地区生产总值增速逐季抬升。“三驾马车”实现稳定增长，全社会固定资产投资2383.65亿元，增长15.0%；社会消费品零售总额2264.10亿元，增长12.1%；进出口总额639.4亿美元，增长4.7%，其中，出口425.3亿美元，增长5.9%。

　　经济结构调整取得实效。三次产业结构比例为2.0：61.9：36.1。以“两化”融合改造提升传统优势产业，搭建“中国在线制造”公共云服务平台，实施“百企携万家登云”计划，全市累计有106家龙头企业、1万多家中小企业上线运行。佛山荣获“中国十大智慧城市”称号。以产业链招商促进工业结构调整，引进北汽福田、中国南车、三全食品等一批大项目。一汽大众项目一期建成投产，带动45家汽配企业进驻，二期正式签约，投资超150亿元。以节能减排倒逼企业转型升级，新增省级清洁生产企业39家，预计万元GDP能耗下降4.25%，主要污染物排放量完成省下达任务。三水区工业园成为国家级分布式光伏发电示范区。实施提升服务业发展水平行动计划，第三产业发展有新突破。欧司朗亚太总部、毕马威大中华区后援中心、慧聪中国家电电子商务产业园、苏宁电子商务运营基地、华侨城文化旅游综合体、华南新加坡城等一批现代服务业项目落户。我市成为省服务外包示范城市。农业现代化进程加快，新增省级以上农业龙头企业5个、农民专业合作社36个。

　　民营经济发展提速。出台推动民营企业跨越发展的政策措施，禅城区、南海区、顺德区分别推出“醒狮计划”、“北斗星计划”和“顺德18条”，三水区实施产业培优工程，高明区建立“企业服务卡”制度。45家骨干企业制定了做大做强计划，9家企业实现产值超百亿元，其中碧桂园集团主营业务收入突破千亿元。

　　产业载体集聚效应凸显。中德工业服务区发展总体规划通过省政府审议，定位为“广东工业服务综合配套改革试验区”，引进史太白技术转移中心、VJP知识产权事务所、F+U教育集团等一批德国工业服务机构。佛山国家高新区核心区与狮山镇实现“园镇融合”，加快打造狮山汽车产业城，做大做强光电、生物医药等新兴产业，辐射带动能力显著增强，去年全省高新区考核跃升至第3名。广东金融高新区获批省金融科技产业融合创新综合试验区，累计引进148个项目，总投资超过393亿元。顺德高新技术产业开发区升格为省级开发区。南方智谷、广东工业设计城、华南电源创新科技园、欧洲工业园、海尔（三水）创新产业园等建设加快，产业集聚效应明显。

　　二、全面推进国家创新型城市建设，创新创造能力提升

　　创新环境得到优化。全市财政投入科技创新资金超过20亿元。以企业为主体的创新体系不断完善。企事业单位申报省级科技计划项目1444项，同比增长14%，新增高新技术企业96家。知识产权战略深入实施。新增中国驰名商标25个，总数达118个；集体商标1件，总数达16件；有效发明专利4537件，增长19.4%；11个项目获第十五届中国专利奖。禅城区成为首批国家知识产权强县工程示范区。

　　产学研合作成果丰硕。与中科院、清华大学、卡内基梅隆大学等院校合作不断深化，新增产学研合作项目370个、成果转化项目240个，实现产业化项目120个，带动产值40多亿元。西安交大广东研究院、广工大数控装备协同创新研究院、佛山中科院产业技术研究院等17个重点创新平台加快发展，佛山火炬创新创业园、南海瀚天科技城成为“国家级科技企业孵化器”。重大关键技术攻关开展顺利，选取铝型材、陶瓷、家电、3D打印、工业机器人领域进行全球技术招标。高端创新人才引进初见成效。新增省级、市级创新团队15个，吸引国家“千人计划”人选6名来佛山投资创业。

　　金融创新成绩显著。金融创新“九个一”工程[1]顺利推进。建成佛山民间金融街，吸引金融、中介机构近50家进驻；广东金融高新区股权交易中心挂牌，注册企业已达242家，融资意向60亿元；新增5家银行机构、21家小额贷款公司、45支股权投资基金。深交所企业上市路演中心进驻南海，天交所广东运营中心落户顺德。设立产业金融引导基金，在全省率先出台小额贷款公司分类管理办法和监管实施细则。美的集团和佛山总商会分别牵头发起设立民营银行申请。中国集成金融集团、海天调味食品有限公司分别登陆香港、上海主板市场，美的集团实现A股整体上市，我市上市企业总数达39家。

　　三、大力推进城市升级，城市形象品质进一步提升

　　城市升级亮点纷呈。《佛山市城市总体规划（2012—2020年）》通过部、省联审，佛山城市中轴线规划通过国际竞赛确定实施方案。中心城区建设和改造提速。禅城老城区中山公园敞开绿化空间，与汾江河景观融为一体，季华路快速化改造及沿线重点项目建设加快推进。禅西新城绿岛湖都市产业区、佛山市外国语（国际）学校等项目动工建设，智慧新城启动区全面建成。佛山新城中德工业服务平台等CBD九大项目加快建设，公共文化综合体主体竣工。千灯湖片区金融公园、灯湖广场等建设顺利，金融高新区配套设施日益完善。实施狮山环境配套提升工程，博爱湖、南海中央公园、南海市民服务中心等项目建设有序推进。顺德新城路（绿）网建设取得进展，43个重点项目完成10个，东拓步伐持续加快，大良、容桂中心区域明显优化。西江新城明湖艺术公园、君御温德姆至尊酒店初步建成，体育中心、文化中心主体工程封顶，成为新城开发的新亮点。三水新城市政道路建设、北江新区拆旧建新同步推进，北江体育休闲公园基本完成。城市升级由点到面成效初显。涌现出佛山新城滨河区、季华商务带、千灯湖片区、西樵听音湖片区、德胜河南岸滨水区、西江新城核心启动区等一批片区景观，以及江湾立交、广珠西线顺德站出入口、顺峰山公园东入口、高明大道海天立交、三水健力宝立交等一批节点景观，佛开、广梧等高速沿线及北江沿线建设生态景观林带126.4公里。中心城区沿街景观综合整治和道路“五位一体”[2]改造同步展开，禅城区松风路，南海区佛平路（军桥—桂澜路段）、博爱路（佛山一环—虹岭路段），顺德区伦教羊大路、新成路、北滘林头村道路，三水区广海大道成为改造样板。北滘等镇（街）绽放小城魅力。“三旧”改造助推城市升级，岭南天地二期对外开放，佛山国际家居博览城开业，伦教霞石村整体改造成效显现。

　　基础设施日益完善。贵（南）广、佛肇城际佛山段征拆工作基本完成。佛山西站、广佛环线动工建设，广佛江珠轻轨开展前期工作，广佛线二期完成工程总投资的15.71%，地铁2号线TOD[3]招标完成，南海区新型公交系统试验段动工建设。广明高速西延线建成通车，广明高速陈村至西樵段工程一期、肇花高速三水段完成投资过半，汾江路南延线、魁奇路东延线佛山段、顺德环城快速干线等一批重点路桥项目建设加快推进，龙湾大桥主线和油金大桥扩建工程建成通车。东平水道航道整治工程基本完工。国家重点项目500KV“西电东送”换顺线工程及东坡输变电工程建设稳步推进，佛山南部、北部电网得到加强。

　　生态环境建设力度加大。出台最严格环境保护考核办法和责任追究制度。开展大气污染综合防治，整治工业锅炉、VOCS[4]企业，推广粤IV车用汽油，启动黄标车抓拍系统，设立黑烟车检测点，实施奖励政策提前淘汰黄标车，更新黄标公交车695辆。落实最严格水资源管理制度。大力推进水口水道、西南涌、芦苞涌等广佛跨界重点河流治理，汾江河告别劣V类水质；启动42条重点内河涌“一河一策”整治，丰收涌、谢边涌等治理成效初显。环保基础设施建设加快，54间污水处理厂投入运营，各区污泥处置设施基本建成。开展国家生态市创建工作，21个镇（街）成为国家级、省级生态乡镇，顺德区杏坛镇逢简村获评全国最美乡村。推进造林绿化工作，获评“全国绿化模范城市”。新建和改造孝德湖公园、海逸社区公园、兆祥公园等一批生态公园。

　　城市管理水平持续提高。城市管理考评实现常态化。深入开展乱摆卖、乱搭建、建设工地不文明施工等城市“乱象”整治行动，重点区域和路段“脏乱差”现象明显改观。稳步推进城市生活垃圾处置工作，“五个五”城市生活垃圾分类试点[5]顺利完成，“一区一场、一镇一站、一村一点”垃圾设施全面建成，城乡生活垃圾无害化处理率达94%。通过“管限引建”[6]推进交通治堵工作，中心城区交通拥堵局部缓解。城市公交线路持续优化，禅城、南海、顺德三区公交对接加快，新增公交线路29条、公交站场13个、公共自行车4000辆、公共停车位7369个，中心城区公交分担率从前年的22.04%提高至23.7%。

　　《规划纲要》实施进入新阶段。“四年大发展”考核位居珠三角第4名，“九年大跨越”[7]工作启动。广佛同城化取得新进展，番顺公路五沙大桥扩建新桥建成通车，珠江大桥放射线接广佛新干线开展招标，西二环乐平互通立交开工建设。广佛肇经济圈建设推进顺利。佛港澳合作进一步深化。佛山新一轮对口帮扶云浮工作正式启动，建立两市联席会议制度，设立对口帮扶指挥部，两市进入携手合作快车道。

　　四、积极推动文化事业繁荣发展，城市文化软实力增强

　　传统文化保护开发不断加强。古椰贝丘遗址、顺德糖厂和清晖园获评第七批全国重点文物保护单位。仁寿寺改造提升进展顺利，祖庙古建筑修复工程、梁园扩建工程设计方案完成。传统文化“走出去”取得良好成效，佛山陶艺文化系列活动在国家博物馆成功举办，佛山粤剧、武术、醒狮赴国外展演均受好评。

　　市民文化生活日益丰富。南海区、顺德区博物馆新馆建成使用，佛山市图书馆新馆、佛山大剧院等一批公共文化设施建设加快，村（居）全民健身工程和“五个有”[8]标准社区文化设施基本实现全覆盖。成功举办2013年秋色欢乐节、佛山龙舟文化节和佛山美食节。“寻梦佛山”异地务工人员子女文化艺术夏令营荣获第十六届全国“群星奖”。“魅力佛山”艺术惠民工程顺利实施，“梦想舞台”成为市民展示艺术才华的圆梦平台。

　　市民文明素质稳步提高。以“微文明·微志愿”活动品牌，推动公民道德教育和文明习惯养成，组织“道德模范巡讲团”活动，开展学雷锋志愿服务，全市注册志愿者近50万人，占建成区常住人口的24.5%。“创文”工作在年度国检中取得良好成绩。

　　五、加快发展社会民生事业，人民群众幸福感增强

　　民生实事圆满完成。公共租赁住房开建4647套，基本建成6494套，超额完成年初目标任务。实施学前教育三年保教费补助制度，惠及学前三年户籍幼儿9.63万人，补贴金额4589万元。实施学前教育户籍在校生生均公用经费财政拨款制度，每生每年100元。全市九年免费义务教育对象扩大到所有在校生（含民办学校）。新建6家健康村（居）。建立大病保险制度，报销最高限额20万元。完成未就业残疾人摸底统计。对本市户籍居民独生子女伤病残或死亡后未再生育（或收养）的夫妻，每人每月扶助金标准分别提高到600元和800元。发放低收入群众物价上涨补贴2421万元，惠及群众近6.5万人次。新增家庭服务中心19个。率先实施生育保险新政策，共核发生育津贴3.08亿元。常住人口免费孕前优生健康检查目标人群覆盖率达99%。户籍人口殡葬基本服务费用全额免除。

　　社会事业加快发展。全市财政民生支出313.94亿元，占公共财政预算支出的64.24%。教育事业再创佳绩。成为首个“广东省推进教育现代化先进市”，在全国率先建立中职、高职、应用型本科教育衔接体系。佛山科技学院新校区动工建设。就业和社会保障工作加强。城镇新增就业9.09万人，城镇登记失业率2.26%，高校毕业生就业率达93.8%，“零就业家庭”保持动态归零。城乡居民一体化社会养老保险体系进一步完善，居民住院医保实现市级统筹，退休职工人均养老金增至2135元/月，失业保险金增至1048元/月，居民住院报销比例达75%。医药卫生体制改革继续深化。政府办基层医疗卫生服务机构全部实施基本药物制度；推行平价医疗服务，设立平价医院、平价诊室和平价药包。佛山市妇女儿童医院动工建设。稳定物价水平，新建平价商店114家。“两建”[9]工作全省领先，在全省考评中位居非试点城市第1名。对口援建和革命老区扶贫“双到”任务全面完成。

　　平安佛山建设成果突出。平安佛山细胞工程建设[10]扎实推进。公安专业化精确打击战略深入实施，流动人口和出租屋专业化管理在全市推广，110刑事警情同比下降22%，社会治安形势持续向好。城市应急综合管理机制进一步完善。安全生产形势稳定，安全生产标准化达标企业增至1.03万家。食品安全示范市建设成效显著，累计完成升级改造农贸市场223个，建成省级市级“菜篮子”基地29个、阳光厨房1483家、食品安全示范点1028家、食品集中加工中心10个，全市实现肉品冷链统一配送。成功举办第二届广东（佛山）安全食用农产品博览会。

　　六、着力推进改革创新，发展动力活力增强

　　行政体制改革呈现新亮点。合并卫生、计生部门，重组市食品药品监管局。调整佛山新城、乐从镇管理体制，委托顺德区管理，实行区镇融合、联动发展机制。佛山网上办事大厅建设全省领先，市、区两级行政审批事项全部进驻，全市可网上申报审批事项达97.34%，全程网上办理事项达24.07%。市民融合服务平台实现1300多项政务服务网上办理，“市民之窗”自助服务终端在禅城、南海成功试用，12345政务平台实现全市热线统一呼叫和指挥调度。企业注册登记改革全面实施，登记时间压缩到7个工作日，初步建立“宽进、快审、严管”企业管理新模式。建设工程联合验收改革实现三级联动，验收时限从约100个工作日压缩到平均7.8个工作日。顺德区商事登记制度改革走在全省前列，在国内率先发布企业经营审批事项目录，南海区在全省率先实现行政审批标准化、程序化和电子化运行。

　　经济体制改革实现新突破。整合集中优势资源，稳步推进政府优债工作。改革企业投资管理体制，实行以备案制为主的项目管理体制。成功举办第九届城市可经营项目投资推介会。引入财政支出项目“过程绩效”管理机制，扩大财政专项资金竞争性分配范围；深化国库管理制度改革，市本级国库集中支付资金比例达100%。实施公共资源交易“管办分离”[11]改革，规范农村“三资”[12]管理。探索统计体制改革，新增“四上企业”[13]入库1199家，个体工商户转企业工作推进顺利。

　　社会组织发展取得新成绩。全市新增社会组织656个，增长18.5%。设立专项扶持资金，建立市、区、镇（街）三级社会组织孵化基地，试行社会组织登记和年检委托制。加强社会组织管理，引进第三方审计机构进行财务监管。建立社区服务中心等服务平台，培育发展“街坊会”、“邻里中心”等社区社会组织，实现民办社工机构五区全覆盖。

　　与此同时，我们进一步加强政府自身建设，自觉接受人大和政协的监督，认真办理代表建议和委员提案，办复率达100%。坚持走群众路线，创新开展政务微博、“微访谈”活动，通过“线上收集、线下解决”方式，广集民意、广汇民智、共谋民利，丰富了民主科学决策机制。严格落实“八项规定”，公开“三公”经费，精减文件、会议，大力整治“四风”，查处了一批违法违纪案件。以打促防，强化廉政风险防控。全面推进政府绩效管理体制改革，促进了各项政策措施落实到位。

　　此外，审计、粮食储备、民族宗教、双拥优抚、外事侨务、台湾事务、档案方志、社会科学、人防、气象等工作取得新成绩。

　　各位代表！过去的一年，我们群策群力，风雨同舟，成绩来之不易。在此，我代表佛山市人民政府向全体市民，异地务工人员，中央、省驻佛山单位，驻佛山人民解放军指战员、武警官兵和社会各界人士表示崇高的敬意！向长期关心支持佛山建设的港澳台同胞、海外侨胞、国际友人表示衷心的感谢！

　　回顾一年的工作，我们清醒地认识到，发展过程中还存在不少困难和挑战，主要表现在：发展方式仍然较为粗放，产业结构调整升级任重道远；资源能源要素制约仍待化解，节能减排形势严峻，大气、水、土壤的污染治理任务艰巨；高新技术产业、现代服务业发展不充分，企业自主创新能力整体不强；中心城市服务功能偏弱，城市规划、建设与管理面临许多新课题；基本公共服务向外来人口全覆盖仍然不足；政府职能转变和作风建设仍需深化。这些问题，需要我们在今后工作中深入研究和切实解决。

2014年工作安排

　　2014年是贯彻落实党的十八大和十八届三中全会精神，全面深化改革的第一年。

　　从外部环境看，世界经济缓慢复苏，国内经济企稳回升，市场信心逐步增强。党的十八届三中全会吹响了全面深化改革的号角，必将激发全国各地抢抓机遇、加快发展。从佛山实际看，产业发展基础扎实、活力足、后劲强，支撑发展的环境和条件优良，经济发展稳步进入“转型升级”、“提质增效”新阶段。特别是十八届三中全会提出加快完善现代市场体系、支持非公有制经济健康发展、深化行政管理体制改革等举措，将有助于我市进一步发挥市场体系较为完善、民营经济较为发达、政府服务高效快捷的综合优势，加快打造佛山经济升级版，实现新形势下新跨越。

　　2014年政府工作的总体要求是：全面贯彻落实党的十八大、十八届三中全会和省委十一届三次全会精神，按照市委十一届五次全会的总体部署，以开展党的群众路线教育实践活动为动力，以建设人民满意政府为抓手，以“加快转型升级、建设幸福佛山”为目标，坚持稳中求进，突出改革创新，着力激发市场活力，加快推进政府职能转变、产业提升、城市升级和环境优化，努力保障和改善民生，切实提高经济发展质量和效益，促进经济持续健康发展、社会和谐稳定。

　　2014年经济社会发展主要预期目标是：全市生产总值增长 10%，人均生产总值增长9%；地方公共财政预算收入增长10%；城镇居民人均可支配收入增长9%；农村居民人均纯收入增长9.5%；居民消费价格总水平涨幅控制在3.5%左右；城镇登记失业率控制在3.0%以内；主要污染物排放指标完成省下达年度计划。

　　实现上述目标任务，我们要重点抓好以下七方面工作:

　　一、全面深化重点领域改革，增创佛山发展新优势

　　发挥佛山善于改革、勇闯新路的传统优势，全方位汇聚全面深化改革的强大正能量，不断优化法治化国际化营商环境。

　　着力推进经济体制改革。以营造更加公平开放透明的市场环境为目标，探索“负面清单”管理模式[14]，降低市场准入门槛，鼓励社会资本进入教育、医疗、文化、体育、交通、通讯等公共服务领域，增加城市优质公共服务市场供给。健全土地、技术、资本、劳动力等要素市场，促进要素市场投资多元化、流动自由化、运作高效化，充分发挥市场在资源配置中的决定性作用。深化企业注册登记改革，降低创业成本,激发社会投资活力。全面实施企业投资管理制度改革，赋予企业更大的自由投资权。积极发展混合所有制经济，以管资本为主加强国有资产监管。深化财政管理体制改革，完善财政专项资金竞争性分配改革，推进“金财工程”[15]建设，试行重大建设项目跨年度预算管理办法。建立政府性债务风险防控和预警机制。健全“两建”工作机制，完成全市社会信用体系建设规划，力争在全省率先建成公共联合征信系统。

　　深化行政管理体制改革。全面推行“三单”[16]管理制度，构建透明公平、监督有力的权力运行新机制。推广行政审批标准化建设，规范政府审批服务，最大限度压缩自由裁量权。以市民融合服务平台为载体，加快实施信息惠民工程，重点加大网上办事服务力度，推进“三网融合”[17]，完善12345政务服务平台，全面提升各级政府公共服务水平和社会管理能力。进一步改革行政执法体制，推进联合执法、统一执法。进一步完善大部门制改革，调整优化机构职能配置，理顺职责关系，加强对基层事权监管和业务指导。推进事业单位改革，做好事业单位法人治理、法定机构试点。

　　积极创新社会治理体制。开展“城乡社区建设提升年”活动，加强城乡社区基础设施“六个一”工程[18]建设，增强城乡社区服务功能，着力构建社区自治体系。推动社会组织完善法人治理机制，激发社会组织活力。充分发挥各级社会组织孵化基地作用，培育发展和规范管理社会组织。完善政府向社会转移职能制度，推广政府购买公共服务。健全社工人才评价办法，引导本土民办社工机构健康发展。支持和发展志愿服务组织。创新有效预防和化解社会矛盾体制，改革行政复议体制和信访工作制度，健全及时就地解决群众合理诉求机制。

　　稳步推进农村综合改革。继续推进村居“政经分离”、“政社分离”改革，加强“两个平台”[19]监管，完善“三资”管理制度，支持有条件的区建立农村集体股权管理交易平台。开展农村土地承包经营权确权登记发证工作，完善土地征收和补偿安置制度，探索农村集体土地直接入市机制，稳步推进农村土地承包经营权流转，保障和发展农民权益。支持南海区进行集体经济产权制度改革，顺德区深度推进基层治理改革，三水区探索村民自治决策、执行、监督三分开。切实做好村（居）两委换届工作。

　　二、推进金融科技产业深度融合，加快构建现代产业体系

　　坚持创新驱动，扎根实体经济，充分激活科技、金融、人才等发展要素，助推佛山产业二次腾飞。

　　创新推动第三产业大发展。把做大做强第三产业作为今年重要工作任务，研究出台更具创新性和突破性的政策措施，推动第三产业发展提速、比重提高、水平提升。大力发展工业设计、文化创意、金融服务、科技信息等生产性服务业，推进跨境电子贸易商务，促进物流、信息等相关配套产业发展；积极发展教育医疗、养老服务、文化娱乐、康体休闲、商务旅游等生活性服务业，发挥佛山中医药文化优势，做大健康产业，打造特色旅游业；稳步推进个体工商户转型升级、公（水）路货运业提升发展；继续开展“万企触电”电子商务行动，促进“广货网上行”。认真落实提升服务业发展水平行动计划，加快广东工业设计城等14个省级现代服务业集聚区及国药集团中药南方总部、苏宁电子商务运营基地、华侨城文化旅游综合体、普洛斯丹灶物流园、王府井商业中心等一批重大服务业项目建设。

　　坚定不移调整优化工业结构。继续实施产业链招商行动计划，着力引进一批先进制造业和战略性新兴产业，促进工业产业向价值链高端发展，向微笑曲线两端延伸，增强工业核心竞争力。加快一汽大众项目二期、北汽福田、南车高明产业基地等一批重大项目建设，形成经济新增长极。大力推进“两化”深度融合，依托“中国在线制造”公共云服务平台，推动装备制造、家电、家具等传统优势产业开展产品、服务、技术、标准、品牌、市场和商业模式创新，促进佛山制造向佛山创造转变。加大节能减排力度，以环境保护倒逼企业转型升级。大力推进“三旧”改造，进一步落实“三旧”改造政策，健全土地增值收益分配机制，完善历史用地手续，加快推进高速公路沿线旧厂房、中心城区旧村居、旧物业等改造，破解产业转型升级的土地瓶颈。做好全国第三次经济普查工作。加大工业反哺农业力度，以农业生态园区为载体，以科技兴农为路径，大力发展优质安全、高效生态农业。创新农业生产经营方式，培育发展农业龙头企业、农民专业合作社等新型经营主体，提高农业生产集约化、专业化、组织化和社会化水平。

　　助推民营经济做大做强。坚持企业需求导向，进一步落实和完善土地、金融、财税、人才等扶持政策，实行“一企一策”，推动一批大型骨干企业产值跨越100亿元、500亿元和1000亿元。以美的集团、一汽大众等重点企业为行业龙头，加强产业链上下游协同创新，进一步提升家用电器、汽车制造等全产业链集群竞争优势。大力扶持中小微民营企业发展，鼓励和引导中小民营企业进行公司制、股份制改造。继续开展高级经理人、企业接班人培训，不断提高企业管理者素质。积极实施“走出去”战略，帮助企业拓展俄罗斯、南美、非洲、东盟等新兴市场。重点支持美的集团、格兰仕集团等骨干优势企业加速海外生产、投资、并购步伐，打造世界名牌。

　　加快国家创新型城市建设。启动“国家创新型城市试点”申报工作。改革政府科技投入体制和评价机制，用好用活企业研发费用税前加计扣除政策。突出企业创新主体作用，建立健全产学研协同创新机制，引导企业与高校、科研院所组建产学研创新联盟，打造一批协同创新中心和公共研发平台；推动企业与中科院产业育成中心、弗劳恩霍夫协会等科研院所、高校开展项目研究和对接，加速科技成果产业转化和应用；推动企业参与国际标准、国家标准等各类标准制定和修订。加强知识产权运用和保护，支持设立知识产权法院，力争建立一个专利技术交易平台。支持禅城区建设“广东省科技服务业集聚区”，南海区建设“广东省金融科技产业融合创新综合实验区”，顺德区建设“广东省自主创新特区”，高明区建设“佛山中科院产业技术研究院新材料产业园”，三水区建设“合肥工业大学广东研究院”。

　　加大金融服务创新力度。依托广东金融高新区集聚发展各类金融创新机构和金融服务产品。创建“广东省民间金融改革发展试验区”，激发民营金融机构创新活力。引导民间资本参与农信社改革、设立民营银行、小额贷款公司、融资性担保公司、融资租赁公司等地方金融机构。积极推广科技保险试点和国家专利保险试点，推动知识产权质押融资，探索区域融资租赁模式，搭建区域集优债和中小企业私募债券发行平台。做好企业上市的各项工作，支持企业股改并参与“新三板”试点。推动符合条件的企业发行公司债、中小企业集合债券、战略性新兴产业集合票据、私募债等融资产品。

　　为产业转型升级提供强大支撑。加快重大平台和产业基地建设。佛山高新区要认真实施产业总体发展规划，加快核心区建设，推进一区五园协同创新发展；建设佛山科技街，促进创新资源汇聚，力争3-5年内进入全国高新区20强。中德工业服务区要深化中德合作，加快聚集德国等欧洲发达国家高端制造业和服务业资源，打造国际化的政务服务体系、产业发展制度和生活配套环境，建设广东工业服务高端国际合作区、广东工业服务综合配套改革试验区。华南电源创新科技园、欧洲工业园为中德工业服务区研发成果提供产业化支撑。继续推进南方智谷、智慧新城、新能源汽车核心部件产业基地、中国（三水）国际水都饮料食品基地、广东新材料产业基地、广东（南海）生物医药产业基地等一批载体建设。夯实创新型城市建设的人才基础。加大人才引进和培养力度，重点引进和培养符合我市发展需求的创新科研团队、创新领军人才、高级技能人才和企业生产管理人才。建立健全科技人才评价标准，创新人才激励政策，进一步放开各类人才入户条件，加快“产业社区”建设，解决人才居住、子女入学、科研、文化娱乐等难题，提升城市凝聚力，以良好的事业平台和优美的生活环境吸引留住人才。

　　三、全面实施产城人融合战略，建设宜居宜商魅力城市

　　坚持走新型城镇化道路，加速推进城市升级和管理，努力打造中心突出、组团清晰、功能完善、生态优美的城市格局，让产业更高端、城市更美丽、人民更幸福。

　　力促城市升级提速增值。突出“强中心”战略，成立中心城区规划建设领导小组，统筹协调跨区域开发建设。禅城老城区要以中轴线规划为统领，力争中轴线北部门户等重要节点开工建设。继续推动祖庙—东华里片区、莲花路—升平路片区等升级改造，形成浓缩岭南历史风情的文化高地。禅西新城要加快佛山市外国语（国际）学校、研发中心、市民广场等优质公共服务设施建设，打造绿色低碳“华南国际化产业集聚区”。佛山新城要抓紧推进CBD九大项目和各项城市功能配套建设，确保商务中心一期、图书馆、科技馆投入使用。千灯湖片区要加快万达广场、广佛智城、广佛国际电商城等项目建设，推动千灯湖特色步行街、虫雷岗山公园等休闲项目改造升级，彰显现代都市风貌。加快推进狮山新城、顺德新城、北江新城、西江新城等组团城市重大项目建设。扎实推进城市轴线和节点更新改造。以水为媒，高标准设计和建设东平河两岸景观灯饰工程，力争打造成为佛山中心城区璀璨靓丽的夜间景观带；以汾江河、绿岛湖、博爱湖、听音湖、德胜河、明湖、云东海等为重点，打造一批环境优美、配套完善的滨河、滨湖生态景观区域。全面建成佛山新城东平水道至乐从、北滘潭洲水道30公里长的滨河景观带。抓好各区主要道路“五位一体”改造，完成禅桂中心区29条沿街建筑景观综合整治。实施佛山市域5条高速公路两侧300米范围内旧厂房改造提升工程，促进城市升级形成整体连片改造效果。

　　打造岭南田园魅力城镇。以田园城市理念[20]为引领，以城乡一体化发展为方向，立足佛山岭南水乡特色，遵循组团城市发展规律，依托南庄、北滘、里水、西樵、白坭、明城等镇（街），建设一批集约、智慧、低碳、绿色田园城镇，努力形成“1+2+5+X” 网络型城乡空间结构。加快城镇土地、产业、人才等要素融合发展，推进城镇教育、医疗、文化等基本公共服务实现均等化，形成“高效率、慢生活”的高品质生活方式。

　　加强基础设施建设。加快广明、肇花、江罗、广中江高速佛山段、佛清从南段、佛江高速公路建设，做好广佛肇高速等项目前期工作。推进各组团间、组团内部交通路网联接，促进组团城市加速融合发展。重点加快中心城区的禅西大道北延线、海五路西延线、绿景路东延线、桂澜路北延线、魁奇路东延线二期、汾江路南延线、岭南大道南延线和顺德区的乐龙路（一期）、南国路东延线、滨河路、海尾立交桥、红旗路（一期）等项目建设。加快广佛线二期工程、贵（南）广、广佛肇城际轨道、南海新型公交系统建设，稳步推进佛山轨道交通2号线一期、广佛环线、佛肇城际轨道7个站点TOD开发建设。加快佛山西站、佛山新城交通枢纽建设。推进城市地下空间的规划和开发利用。抓好佛山新港码头、澜石口岸码头搬迁工作。加强能源、水利、通信、智能电网建设，推进容桂站等一批220KV输变电项目建设，确保国家重点项目500KV“西电东送”换顺线II期工程和500KV东坡输变电工程如期投产。推动光纤入户工程，做好4G基站布局和服务推广。

　　狠抓城市综合管理。重点加强城市道路环境卫生和绿化管养提升、建设工地和泥头车污染源头管控、户外广告清理和违法建设整治，全面提升城市的整洁度、美观度。落实城市管理考核新机制，实行暗检为主、明检为次，推动城市管理工作上新水平。进一步整合数字城管资源，打造智慧城市综合管理数据库中心，促进各区数字城管资源联通共享，提高监管效率和水平。开展给水、排水、燃气、电力、电信等城市地下管线专题数据普查，促进数字化地下管线规划建设。加快中心城区公交一体化进程，推进智能公交建设，统筹优化公交资源和线网布局，提高线路覆盖率。

　　推动区域一体化发展。实施《佛山市推进区域合作工作方案》，统筹推动五区协调创新发展。扎实开展对口帮扶云浮工作，创新帮扶机制，突出产业对接重点，在基础设施、产业园区、新城建设等多领域开展全方位对口帮扶，推动云浮振兴发展、跨越发展。落实“九年大跨越”工作方案，加快推进48个重大项目建设。积极推动广佛同城化，抓好地铁线网规划对接、佛陈路东延线接番禺新桂路（海华大桥工程）、魁奇路东延线接番禺南大干线、三山长江路对接广州南浦大道等工作。推动广佛肇经济合作区建设发展。加强与广州南沙、深圳前海、珠海横琴等国家级平台对接合作。落实好粤港、粤澳合作框架协议，进一步加强现代服务业合作，推动佛港澳服务贸易自由化。把握珠江-西江经济带开发契机，加强与广西、东盟地区的经济互动。

　　四、全力推进生态文明建设，打造绿色美丽家园

　　实施低碳绿色发展战略，加快生态文明建设，构建系统完整的生态文明制度体系，推动佛山实现更长远、更持续、更高质量的发展。

　　优化城市功能布局。按照新型城镇化要求，进一步优化我市城镇化规划布局和功能，因地制宜，分类指导，稳步推进城市开发建设，提高城镇化发展质量和水平。加快建立生态建设统筹推进机制和综合管理制度，严格功能区分类管理，推动各区按照主体功能定位进行开发建设和生态保护。加大对限制、禁止开发区域的保护力度，在高明区探索生态保护区试点。促进资源节约集约利用，支持顺德区开展省城乡土地生态利用制度综合改革试点工作，为全市土地节约集约利用探索新路径。

　　强化生态环境综合治理。做好国家环保模范城市复核及国家生态市创建工作，积极申报国家级生态乡（镇）、村。落实最严格的环境责任考核，强化企业主体责任，全力推进水、气、土壤等环境综合治理。推行“河长制”[21]，实施“一河一策”生态修复、景观提升工程，抓好广佛跨界重点河涌及42条重点内河涌的综合整治。落实大气污染防治“国十条”[22]，通过调整产业布局、强化污染物指标约束、遏制扬尘污染、整治黑烟车、淘汰黄标车、推广应用新能源汽车等措施，缓解PM2.5、PM10[23]等区域性大气污染问题，改善空气质量。今年要完成所有现役燃煤发电机组脱硫改造。全面开展土壤环境调查，加强农业面源污染控制和养殖场污染治理。强化危险物品产生和经营单位管理，加快建设综合性危险废弃物处置中心。建立健全跨区域生态环保联动协调机制，提升跨区域大气、水污染联防联治水平。

　　加快低碳绿色发展步伐。优化能源结构，加快低碳能源管网规划和建设，扩大低碳能源使用范围。抓好全国电力需求侧综合管理试点城市建设，推广三水分布式太阳能光伏发电应用项目，推进电能替代工程，促进能源消费结构转型。发展绿色建筑，全面完成80万平方米绿色建筑建设，开展大型公共建筑能效测评和标识试点。试行碳排放权、排污权交易，完善环境资源有偿使用的市场机制。在陶瓷、家电试点行业，推广碳标志等低碳认证制度。深化禅城、顺德低碳示范区建设，支持佛山新城申报国家绿色生态示范城区。开展低碳教育，创建低碳社区。全面开展新一轮绿化佛山大行动，建设国家森林城市。推进佛山植物园建设。

　　推进生活垃圾减量化。完善垃圾设施建设，改进垃圾分类收运体系，优化垃圾处理工作流程，加快建设有害垃圾终端处理设施或储存中心，逐步实现生活垃圾减量化目标。加大垃圾分类宣传力度，让垃圾分类理念深入人心。大力发展和规范垃圾处理行业，推广狮山环保产业园经验，打造垃圾处理高效产业链。

　　五、深入实施文化强市战略，打造岭南文化名城

　　千方百计加快文化事业大发展大繁荣，让文化发展与经济建设相互促进、比翼齐飞，显著提升佛山城市软实力。

　　加强本土文化资源开发利用。扎实推进仁寿寺、梁园改造提升，加快图书馆现址改造和祖庙古建筑修复工程建设。依托梁园等历史建筑改造，充分挖掘提升中医药和粤剧文化，在汾江河两岸逐步形成佛山古镇风情浓郁的岭南传统文化、商业聚集区。加大对陶塑、武术、剪纸、木版年画等传统文化资源的研究创新，促进非物质文化遗产的保护与传承。

　　健全现代公共文化服务体系。深入实施“魅力佛山”艺术惠民工程，大力引进高雅艺术展演。不断扩大“筑梦佛山”文化艺术公益夏令营受益面，开展“开心广场·百姓舞台”公益流动演出活动，努力丰富市民文化生活。办好第八届市运会，推动群众体育发展。加强公共体育设施和村居全民健身工程的建设和管理，加快亚洲龙舟联合会总部及洲际龙舟广场建设，今年内建成世纪莲游泳馆户外游泳池并对外开放。

　　大力发展文化产业。推动文化与科技、金融融合，扶持文化产业重点园区建设，大力发展工业设计、陶瓷工艺、传媒影视、动漫游戏等文化产业。促进文化与体育、旅游相结合，整合祖庙、仁寿寺、南风古灶、梁园、西樵山、清晖园、黄飞鸿纪念馆、李小龙纪念馆等旅游资源，打造一批旅游精品路线。继续办好佛山秋色欢乐节、佛山龙舟文化节、佛山陶瓷艺术节、佛山美食节。支持南海国家旅游产业集聚（实验）区和佛山文旅街建设，以“一街两脉”[24]文旅轴线为支撑，努力打造佛山文旅绿芯和岭南文旅第一极。

　　着力提升佛山城市知名度。坚持政府主导、市场运作、社会参与原则，鼓励民间文化交流，组织佛山历史文化遗产和当代文化艺术精品到国内外巡回展览（演），推动佛山文化产品和服务“走出去”。加强媒体推介和新闻统筹力度，将文化、旅游、经贸、科技、外事侨务对外资源与佛山城市形象宣传有机结合，多形式、全方位开展城市形象推广，在海内外进一步提升佛山的知名度。

　　力争“创文”通过国家测评。今年是全国文明城市创建的总测评年。要进一步强化创文工作“一把手”工程，以建设“四品佛山”[25]为抓手，全面动员，针对重点任务和薄弱环节，强化巡查督办和考评问责机制，围绕社会主义核心价值观的宣传教育，大力抓好社区志愿服务制度化、“讲文明树新风”公益广告宣传、文明旅游、未成年人思想道德建设等重点工作，营造浓厚的社会氛围，显著提升市民道德素质和社会文明程度，全力冲刺“创文”大考，力争获评“全国文明城市”。

　　六、着力保障和改善民生，确保社会和谐稳定

　　坚持以人为本，更加突出富民、惠民、利民导向，持续提升市民幸福感与社会和谐度。

　　推进基本公共服务均等化。切实抓好教育综合改革试点，深入实施素质教育，打造佛山特色德育品牌。实施学前教育提升计划，统筹城乡义务教育资源均衡配置，全面提升普通高中发展水平，深化职业教育专业发展，推进特殊教育优质内涵发展。加快佛山科技学院新校区建设。完善医疗卫生服务功能，巩固平价医疗服务成果，鼓励非政府办基层医疗卫生机构实施基本药物制度，稳步推进公立医院改革，完善合理分级诊疗模式，提升基层医疗机构服务能力。高标准建设佛山市妇女儿童医院。探索门诊医保跨区就医办法，完成全市户籍人口和在我市参保的异地务工人员社保卡发放工作。指导和帮助高校毕业生、就业困难人员等群体就业，鼓励创业带动就业，全面推进以企业为主体的技能提升培训工作。进一步完善预防和处置企业欠薪工作机制，切实保障劳动者合法权益。推进社会福利和养老服务，加快公办及民营养老福利机构发展，试点实施民办养老福利机构承接政府供养对象收养工作。不断完善社会救助体系，科学调整最低生活保障标准。推进新粮食储备库建设。做好新疆伽师、西藏墨脱对口援建工作。

　　促进异地务工人员市民化。完善流动人口服务管理机制，逐步推进异地务工人员基础服务和基本保障“底线均等”，健全异地务工人员入户、就业扶持、子女入学等优惠政策，让异地务工人员共享改革发展成果，热爱佛山、融入佛山、扎根佛山。充分发挥工青妇、红十字会等群团、慈善组织优势，培育异地务工人员社会服务中心，定期开展主题系列活动，为异地务工人员提供扶贫济困、社交婚恋、法律维权、技能培训等服务。

　　深入推进平安佛山建设。落实平安创建三年行动计划，推广专业化精确打击多发性犯罪模式，确保社会大局稳定。抓好“全国安全社区”和“国际安全社区”创建工作，提升市民安全感。做好国防动员、双拥、优抚安置工作，巩固军政军民团结。健全社区矫正制度，加强特殊人群管理。落实安全生产“一岗双责”，强化企业安全生产标准化和基层“网格化”安全监管体系。推进食品安全示范市建设，扶持“菜篮子”基地、“智慧菜篮子”项目建设，健全农产品交易市场和流通网络，完善鲜活农产品储藏、加工、运输和配送等冷链物流系统，构建食品农产品安全监管社会共治格局。

　　着力办好一批民生实事。加强保障性住房建设，今年新开工建设公租房3360套，基本建成8339套。计划投入6.48亿元建设水环境治理等13个水利项目。加强污水处理厂配套管网建设，今年建设130公里。实施学前教育生均公用经费制度向全市常住人口子女全覆盖。城乡同步实施免费中等职业教育。推进重度残疾儿童“送教上门”。加快残疾人康复工程中心建设。试点开展家庭医生式服务，各区至少建立1个市级示范点，为签约居民提供免费基本公共卫生服务和个性化医疗保健服务。加强公办养老床位建设。实施旅游富民工程，鼓励开办农家乐等乡村旅游项目，在景区评A、饭店评星等方面予以政策支持。全面启动“市民之窗”网上办事自助服务终端项目，确保全市四级行政服务中心布设“市民之窗”终端设备。加大对高明革命老区和三水南山镇的扶持力度，抓好高明苗村及附近区域铺设自来水供水管网建设，加快推进三水迳口华侨农场危房改造。

　　七、深入开展群众路线教育实践活动，建设人民满意政府

　　坚持密切联系群众，尊重人民主体地位,把人民满意作为政府建设的试金石，全面推进“六个政府”建设。

　　建设民生政府。强化可持续发展理念，着力提升发展质量和效益，将发展成果更多更公平地惠及民生。完善经济社会发展成果考核评价体系，加大资源消耗、环境损害、生态效益等指标权重。建立政民互动大平台，让群众参与政府治理。推动新闻发布制度化，健全“微访谈”机制，建立改革发展市民建言献策平台，整合民生直通车、12345政务平台等现有渠道，线上线下问计民生、汇聚民意，纾解民困，形成全方位、常态化的政民互动体系。

　　建设高效政府。坚持问题导向，扎实开展调研，找准改革突破口，切实解决实际问题。着力提升政府执行力，推进重点工作“项目化”，做到“任务、时限、责任人、要求、措施”五落实。改革行政审批制度，全面推行“五个一”[26]服务模式，促进治理水平现代化。深化绩效管理体制改革，通过绩效管理与考核挂钩促进政府治理创新。

　　建设法治政府。坚持依法行政，维护法律权威,依法接受人大法律监督、政协民主监督。落实《佛山市重大决策程序规定》，建立健全市政府重大决策运行机制，将公众参与、风险评估、专家咨询、合法性审查和集体讨论作为重大决策的必经程序，促进科学、民主、依法决策。加强基层执法队伍建设，强化行政执法监督。加强普法宣传和舆论引导，重点推进领导干部学法用法常态化、制度化。推进权力公开透明运行，深化政务信息公开，重点抓好预决算、“三公”经费公开。

　　建设责任政府。按照权责一致原则，推行权力清单工作制度，有序推进各级政府及其部门权力清单制定和公布，明确权责主体和关系。试点实施岗位职责说明书制度，把行政责任落实到具体岗位。建立政府部门行政问责、行政过错责任追究和工作绩效公开制度，接受社会和舆论监督。

　　建设诚信政府。加快政务诚信体系建设，编制政务诚信征信目录，开发政务诚信信息平台。保障重大政策、规划和重点工作的延续性，坚决兑现对群众、企业和投资者的各项承诺，以实际行动取信于民。培育诚信政务文化，增强各级政府、部门及其公务员的诚信意识。以政府诚信带动社会诚信，营造“守信为荣，失信可耻”的社会环境。

　　建设廉洁政府。深入开展廉洁从政教育，坚持用制度管权管事管人，推进预防腐败机制创新，把权力关进制度的笼子里。加强行政监察、审计监督和对重点企（事）业单位廉政风险防控。深化公共资源交易改革，提升对招投标领域的有效监管水平。加强对权力的网络监督，完善网络舆情反馈处理机制。严厉惩处失职渎职、贪污贿赂行为。贯彻党风廉政建设责任制，认真落实“八项规定”，纠正“四风”，着力整治庸懒散奢，减少文山会海，健全改进作风常态化制度。

　　各位代表！新一轮改革的春天已经来到，未来发展的蓝图已经绘就。让我们在党的十八大和十八届三中全会精神指引下，在省委、省政府和市委的坚强领导下，解放思想、抢抓机遇、改革创新、攻坚克难，为开创佛山改革发展新局面而努力奋斗！
